# The changing face(book) of psychiatry: can we justify ‘following’ patients' social media activity?

**DOI:** 10.1192/pb.bp.114.049130

**Published:** 2015-12

**Authors:** Chantal Cox-George

**Affiliations:** 1 Bristol University, UK

## Abstract

Individuals with mental health issues may post information on social networking sites that can provide an insight into their mental health status. It could be argued that doctors (and specifically psychiatrists) should understand the way in which social media is used by their patients to gain a better insight into their illnesses. However, choosing to actively monitor a patient's social media activity raises important questions about the way in which medical students, qualified clinicians and other healthcare professionals obtain information about patients. While this may be framed as a mere form of ‘collateral history-taking’, there are obvious practical and ethical problems with doing so. Here, a case is made against monitoring the social media activity of patients involved with psychiatric services.

In 2013, nearly one in four people worldwide actively used social networking sites, a statistic predicted to increase rapidly throughout this decade.^1^ Given that so many people are using social networking sites, it may be no surprise to find that many people with mental health issues have a social media presence too. For example, there are a plethora of ‘pro-ana’ (pro-anorexia) websites, blogs and Facebook groups in existence for individuals with eating disorders. These have proven to have both therapeutic and potentially dangerous effects on illness behaviour.^2,3^

People with mental illness may post information online that provides an insight into their current mental health. If this is the case, then doctors (and specifically psychiatrists) should understand the way in which social media is used by patients as it may allow them to gain a better insight and, subsequently, provide better care.

To explore these premises, I consider the act of looking at a patient's Facebook page, Twitter activity or personal blog as merely another form of ‘collateral history-taking’. Focusing specifically on the Facebook ‘status update’ – a way in which individuals may post their current thoughts and feelings (with a time and date stamp) – I ask whether this is a way to access a patient's mental state in real time. Given that the majority of Facebook profiles are public (meaning that the profile owners have chosen not to opt into privacy settings), any updates posted are available to not only Facebook ‘friends’ but also others within the person's associated ‘networks’ and those outside, for instance healthcare professionals.

A study carried out in the USA aimed to assess the prevalence of college students' disclosures of depression symptoms on Facebook.^4^ Despite the potential for stigma surrounding mental health symptoms or diagnoses, a quarter of profiles observed publicly displayed depression references. However, should we take this statistic seriously? We may, wrongly, be talking about an overrepresented population. It might be that patients with particular mental health conditions or certain personality types are more likely than others to frequent the likes of Facebook, Twitter and other forms of social media more often. It has been suggested that there may even be a correlation between excessive internet use and social anxiety, depression and introversion.^5^ Furthermore, we cannot be sure that the information posted in an update is accurate. Creating a social media profile allows profile owners to be selective about the aspects of their identity they wish to display and those they wish to avoid putting into focus. The ‘online disinhibition effect’^6^ states that when people are online, they tend to disclose more about themselves or act out more intensely or frequently than they would in person. This suggests that we should exercise a degree of caution when considering information posted online.

Nonetheless, even if the information posted online by individuals with mental health issues is accurate, there appears to be a ‘fine line’ between monitoring and being meddlesome. Once a doctor has demonstrated that their actions would be of benefit to the patient, the most pressing question to consider next is whether they should ask the patient for their consent. The Human Rights Act 1998 states that everyone ‘has a right to respect for his private and family life, his home and his correspondence’. This applies even if seeking a patient's consent will have an effect on their future activity online. The obvious response to this is that the information has already been made public, and so patients have waived any rights that they had to privacy. Yet, a survey of 492 bloggers demonstrated that people often disclose information online with a particular audience and time period in mind, even though the information may then become broadly available for an indefinite period.^7^ Medical students and qualified clinicians should be aware that accessing a patient's social networking profile through covert and unauthorised means may form a basis, at least in the patient's opinion, for the argument that they have infringed upon their patient's private life.

Finally, if we proceed without consent and the patient finds out, there may be serious effects on the psychotherapeutic relationship: a relationship based on the active engagement of the patient which can no longer happen if the patient does not trust the healthcare professional. This is likely to have implications for the patient's health. There is an implicit understanding that a patient's trust in their doctor is unconditional. In response to the new dilemmas that may arise in clinical practice due to the rise of social media use by both patients and clinicians alike, the General Medical Council has reiterated the importance of trust not only in a doctor's clinical practice but also in their online behaviour.^8^

If a psychiatrist takes it upon themselves to do further research on their patient online and finds conflicting information, it is difficult to see how this could be used without challenging the patient's narrative. This is further complicated by the question of what to do with any new discoveries about the patient that may surface. Options for the psychiatrist may include: documenting new information in the patient's notes, conferring with colleagues, telling the other members of the multidisciplinary team involved in the patient's care, or disregarding what they have seen for fear of future repercussions. It is, however, important to consider whether the psychiatrist has a duty of care to act on information of which they would have otherwise been unaware.

While not specifically social media, the internet has been used as part of risk assessment in accident and emergency settings before. The information obtained from a Google search proved to be crucial in a doctor's decision to classify a patient as high- rather than low-risk for future suicidal intention.^9^ However, it would be a slippery slope to suggest that one success justifies following the social media activity of all of our patients. Whereas in theory actively looking at a patient's social media profile might be advantageous, in reality it is unethical (particularly without consent). If doctors plan to use any information found for treatment, then they will have to disclose their intentions to patients before they do so.
